# A Comparative Analysis to Dissect the Histological and Molecular Differences among Lipedema, Lipohypertrophy and Secondary Lymphedema

**DOI:** 10.3390/ijms24087591

**Published:** 2023-04-20

**Authors:** Julia von Atzigen, Anna Burger, Lisanne Grünherz, Carlotta Barbon, Gunther Felmerer, Pietro Giovanoli, Nicole Lindenblatt, Stefan Wolf, Epameinondas Gousopoulos

**Affiliations:** 1 Department of Plastic Surgery and Hand Surgery, University Hospital Zurich, 8091 Zurich, Switzerland; julia.vonatzigen@usz.ch (J.v.A.); anna.burger@usz.ch (A.B.); lisanne.gruenherz@usz.ch (L.G.); carlotta.barbon@usz.ch (C.B.); nicole.lindenblatt@usz.ch (N.L.); stefan.wolf2@usz.ch (S.W.); 2 Division of Plastic Surgery, Department of Trauma Surgery, Orthopaedics and Plastic Surgery, University Medical Center Göttingen, Georg-August-University, 37075 Göttingen, Germany; gunther.felmerer@med.uni-goettingen.de

**Keywords:** lipedema, lipohypertrophy, secondary lymphedema, adipose tissue disorders, CD4+ cells, macrophages, lymphatic vessels, VEGFD

## Abstract

Lipedema, lipohypertrophy and secondary lymphedema are three conditions characterized by disproportionate subcutaneous fat accumulation affecting the extremities. Despite the apparent similarities and differences among their phenotypes, a comprehensive histological and molecular comparison does not yet exist, supporting the idea that there is an insufficient understanding of the conditions and particularly of lipohypertrophy. In our study, we performed histological and molecular analysis in anatomically-, BMI- and gender-matched samples of lipedema, lipohypertrophy and secondary lymphedema versus healthy control patients. Hereby, we found a significantly increased epidermal thickness only in patients with lipedema and secondary lymphedema, while significant adipocyte hypertrophy was identified in both lipedema and lipohypertrophy. Interestingly, the assessment of lymphatic vessel morphology showed significantly decreased total area coverage in lipohypertrophy versus the other conditions, while VEGF-D expression was significantly decreased across all conditions. The analysis of junctional genes often associated with permeability indicated a distinct and higher expression only in secondary lymphedema. Finally, the evaluation of the immune cell infiltrate verified the increased CD4+ cell and macrophage infiltration in lymphedema and lipedema respectively, without depicting a distinct immune cell profile in lipohypertrophy. Our study describes the distinct histological and molecular characteristics of lipohypertrophy, clearly distinguishing it from its two most important differential diagnoses.

## 1. Introduction

Lipedema, lipohypertrophy and secondary lymphedema present three distinct chronic conditions with similar phenotypes, leading frequently to misdiagnosis. The most prominent overlapping feature is the increased adipose tissue deposition in one or more extremities accompanied or not by swelling. The disproportional adipose tissue increase may often lead to decreased range in motion and physical restraints, as well as a psychological burden for the affected patients.

Lipedema is characterized by the disproportionate symmetrical and bilateral deposition of painful adipose tissue affecting mostly the lower limbs, while sparing the feet and hands [1,2]. Further defining features include an increased tendency to bruise, joint hypermobility and the possibility of edema formation in the last stage of disease, defined as lipo-lymphedema. The condition is present almost exclusively among women, with only anecdotal reports of lipedema found in men. It is considered to be triggered by hormonal changes such as puberty or menopause, while the possible mechanisms involved remain elusive. Lipohypertrophy is manifested with the same phenotype as lipedema, without the clinical sign of pain, thus being frequently considered as stage 0 of lipedema. The data supporting such a notion are still missing [1,2]. As far as secondary lymphedema is concerned, it is encountered in the Western world as one of the most relevant complication following oncologic surgery upon removal of one or more lymph nodes and/or radiotherapy, thus affecting both men and women. It is a chronic and progressive disorder as well, characterized by the progressive swelling of the affected extremity and the fibroadipose tissue deposition. The edema distribution usually affects the hands or the feet too. The latter is also characterized by a positive Stemmer sign, which is negative in the other two conditions. The sign is verified with a physical examination; if the examiner can’t pinch the dorsum’s foot or hand’s skin, the sign is considered positive [3].

While the clinical differentiation among the three conditions is mostly straightforward in experienced hands, based on the patient history and clinical examination, our knowledge about the histological and molecular determinants of lipohypertrophy remains restricted. In contrary, the pathophysiology of lipedema and secondary lymphedema has lately been a matter of intense investigation, unraveling key players in the onset and development of the disease. Recent work has demonstrated the absence of structural lymphatic vessel deficits as well as the increased infiltration with M2-polarized macrophages in lipedema [4,5,6]. On the other hand, the changes in the structure and function of the lymphatic system during the course of lymphedema have been well-examined and extensive research by various laboratories across the globe, confirming the causal implications of the increased CD4+ infiltration that is observed in lymphedema [7,8,9,10,11].

Thus far, no information exists regarding immune cell infiltrates in lipohypertrophy and even though the phenotype of lipohypertrophy is described to be very similar to that of lipedema with the exclusion of pain and discomfort [1,12], it remains unclear if any edema formation ever takes place. The hypothesis, that lipedema may progress from lipohypertrophy is tempting, but the current knowledge does not substantiate such a statement [1,2,12]. In fact, our knowledge around lipohypertrophy is scarce and the condition is only briefly mentioned in some national lipedema guidelines as an important differential diagnosis (German [13], UK [14], Dutch [15], USA [16]).

Lipedema, lipohypertrophy and secondary lymphedema are chronic disfiguring conditions, often mistreated as mere adiposity. Initially these conditions are treated conservatively and the patients seek surgical interventions to mitigate the persistent symptoms [16,17,18,19,20,21]. While the interventions to improve lymphedema are constantly gaining acceptance fueled by the increasing body of data and promising clinical outcomes, the surgical treatment of lipedema and particularly lipohypertrophy with liposuction is still often considered an esthetic treatment instead of disease management, restricting the access to adequate treatment [22]. Thus, it is clinically relevant and important to gain deeper understanding of these diseases to ensure their better diagnostic and monitoring methods.

For this purpose, in this study we compared histological and gene expression features of lipedema, secondary lymphedema and lipohypertrophy using gender-, age-, BMI- and anatomically-matched skin and fat tissue from healthy control patients. We confirmed well-known hallmarks of lipedema and secondary lymphedema such as adipocyte hypertrophy, increase in epidermis thickness, fibrosis and immune cell infiltrate changes and analyzed these findings in lipohypertrophic tissue as well, describing its distinct nature and making this the first comprehensive comparative study of the three conditions combined. 

## 2. Results

### 2.1. Epidermis Thickness Is Increased in Lipedema and Secondary Lymphedema Patients, While Cutaneous Fibrosis Is Reduced in Lipohypertrophy Patients

**Figure 1:**
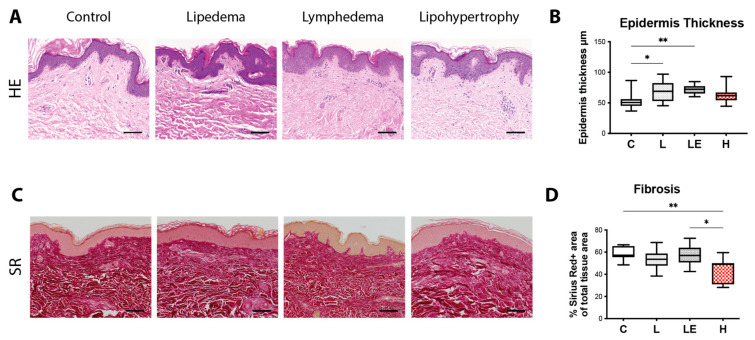
Assessment of the skin architecture and fibrosis content in lipedema, secondary lymphedema and lipohypertrophy patients compared to the control group. (**A**) Representative Hematoxylin/Eosin (H/E) staining of skin from control, lipedema, secondary lymphedema and lipohypertrophy patients. (**B**) The quantification of epidermal thickness across all four groups, depicted increased epidermal thickness in the skin of lipedema and secondary lymphedema patients compared to the control group. (**C**) Sirius Red (SR) staining of the skin was used to evaluate the extend of fibrosis in control, lipedema, secondary lymphedema and lipohypertrophy patients. (**D**) The analysis of the collagen deposition indicated significantly lower levels of fibrosis in lipohypertrophy. Black scale bar represents 100 μm. N = 10 in each group. Statistical significance is signified by asterisks (* *p* < 0.05, ** *p* < 0.01).

In our study we compared skin tissue biopsies collected intraoperatively and stained with Hematoxylin/Eosin to evaluate the skin architecture in lipedema, secondary lymphedema and lipohypertrophy patients to matched controls. Hereby epidermal thickness was found increased in both lipedema and secondary lymphedema patients but not in lipohypertrophy patients, when compared to healthy controls (C = 53.51 ± 14.39 μm, L = 73.63 ± 11.35 μm, LE = 71.95 ± 7.712 μm, H = 64.94 ± 14.66 μm) (Figure 1A,B). Skin fibrosis was assessed by quantification of collagen in Sirius red stainings, which showed significantly lower levels in lipohypertrophy compared to control and lymphedematous tissue. (C: 51.25 ± 6.99%, L: 54.82 ± 6.48%, LE: 56.68 ± 9.25%, H: 43.42 ± 9.95%) (Figure 1C,D).

### 2.2. Adipocyte Size Is Increased in Lipedema, Secondary Lymphedema and Lipohypertrophy Patients

**Figure 2:**
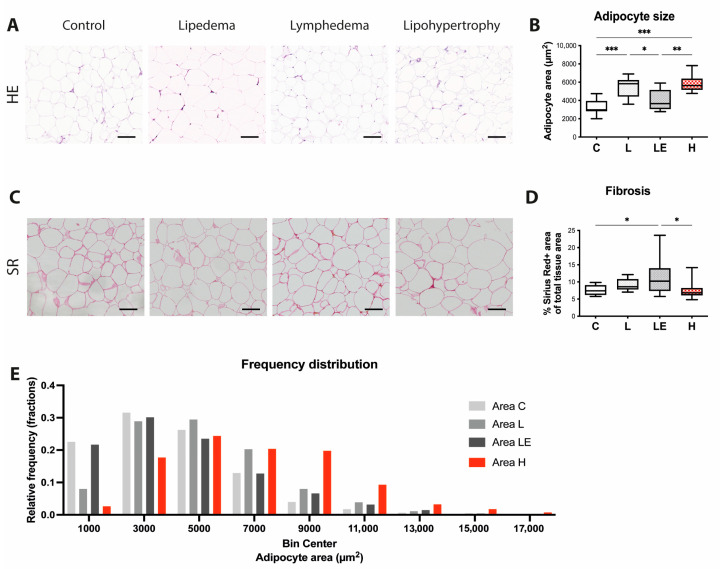
Adipocyte size was significantly increased in lipedema and lipohypertrophy. (**A**) Hematoxylin/Eosin (H/E) staining of adipose tissue in all four groups (control, lipedema, secondary lymphedema and lipohypertrophy). (**B**) Evaluation of adipocyte size comparing all four groups. Significantly larger adipocytes were found in lipedema and lipohypertrophy when compared to the control tissue and secondary lymphedema. (**C**) Sirius Red (SR) staining of adipose tissue in all four groups (control, lipedema, secondary lymphedema and lipohypertrophy). (**D**) Fibrosis analyzed by collagen content comparing all four groups showed significant increase in secondary lymphedema. (**E**) Adipocyte size distribution analysis identified increased number of larger adipocytes in lipedema and lipohypertrophy. Black bar represents 100 μm. N = 10 in each group. Statistical significance is signified by asterisks (* *p* < 0.05, ** *p* < 0.01, *** *p* < 0.001).

Next, we analyzed the adipose tissue architecture in adipose tissue biopsies. Hematoxylin/Eosin stainings were used to assess the adipose size, while Sirius red stainings were used to quantify the intercellular fibrosis. Adipocyte size was found significantly increased across all three conditions when compared to controls, with the level of hypertrophy being comparable between lipohypertrophy and in lipedema, and higher than in secondary lymphedema. (C = 3412 ± 853 μm^2^, L = 5446 ± 1109 μm^2^, LE = 4088 ± 1159 μm^2^, H = 5904 ± 1033 μm^2^) (Figure 2A,B). By analyzing the adipocyte size distribution, we identified a skewed distribution with higher numbers of larger adipocytes in the lipedema and lipohypertrophy tissue compared to control tissue. (Figure 2E). The quantification of the adipose tissue fibrosis showed increased collagen deposition among the adipocytes in secondary lymphedema tissue compared to control and lipohypertrophy tissue (C: 7.67 ± 1.42%, L: 9.12 ± 1.68%, LE: 11.35 ± 4.99%, H: 7.52 ± 2.50%) (Figure 2C,D).

### 2.3. Lymphatic Vessel Coverage Is Significantly Lower in Lipohypertrophy Patients and VEGF-D Expression Is Significantly Decreased across All Conditions, in Comparison to Controls

**Figure 3:**
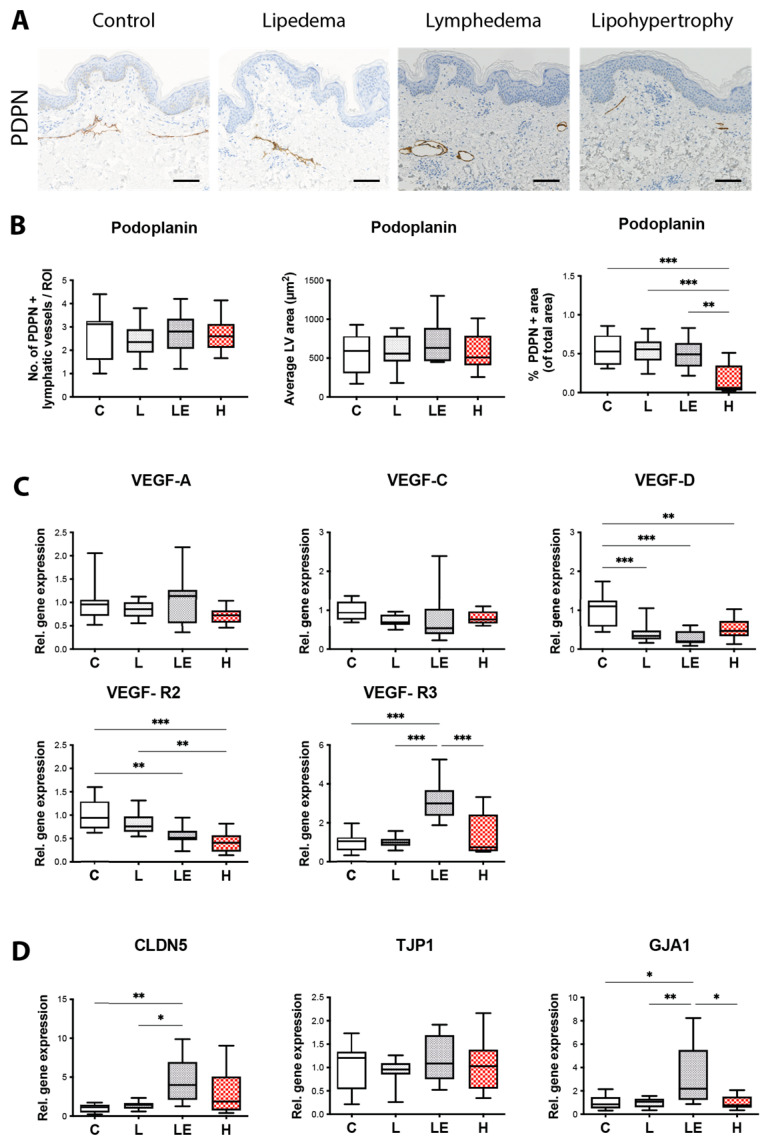
Evaluation of the lymphatic vascular milieu. (**A**) Representative Podoplanin (PDPN) staining of skin sections from all four groups (control, lipedema, lipohypertrophy, secondary lymphedema). (**B**) Quantification of lymphatic vessels by number of vessels, size of vessels and percentage of surface area covered by vessels. A significant decrease in surface area covered by lymphatic vessels was detected in lipohypertrophy in comparison to control group. (**C**) Relative gene expression (hereby control group = 1) of VEGF-A, VEGF-C and VEGF-D. The expression of VEGF-D is significantly decreased across all three conditions in comparison to the control group. VEGF-R2 is significantly decreased in secondary lymphedema and lipohypertrophy when compared to control tissue. The expression levels of VEGF-R2 in lipohypertrophy are significantly lower when compared to lipedema as well. VEGF-R3 expression was significantly increased in secondary lymphedema when compared to all groups. (**D**) Relative gene expression (hereby control group = 1) of GJA1, TJP1 and CLDN5. In GJA1 and CLDN5 expression was significantly increased solely in secondary lymphedema. No change in expression was detected in TJP1. Black bar represents 100 μm. N = 10 in each group (gene expression of CLDN5, TJP1 and GJA1 in LE group = 5). Statistical significance is signified by asterisks (* *p* < 0.05, ** *p* < 0.01, *** *p* < 0.001).

To evaluate possible differences in regard to the number and tissue coverage of lymphatic vessels, skin sections of the three different conditions and control patients were stained with Podoplanin (PDPN). Although neither the number nor the average size of vessels showed any changes across the three different conditions when comparing to controls (Nr: C = 2.625 ± 1.316, L = 2.417 ± 0.8137, LE = 2.747 ± 0.9248, H = 2.702 ± 0.7287; Size: C = 539.4 ± 266.4 μm^2^, L = 597.3 ± 212.4 μm^2^, LE = 711.8 ± 296.7 μm^2^, H = 580.2 ± 245.8 μm^2^), the total lymphatic vessels coverage was significantly decreased in lipohypertrophy when compared to lipedema, secondary lymphedema and matched control tissue (C = 0.6641 ± 0.4228%, L = 0.5157 ± 0.2294%, LE = 0.4971 ± 0.2001%, H = 0.1562 ± 0.1784%) (Figure 3A,B). 

To evaluate whether the lymphatic phenotype observed might be related to modified expression of a vascular endothelial growth factor (VEGFs), the expression of the three most common VEGFs, namely VEGF-A, VEGF-C and VEGF-D, was analyzed (Figure 3C). While VEGF-A and -C did not significantly differ relative to control tissue (VEGF-A: L = 0.8411 ± 0.1795 fold, LE = 1.103 ± 0.5908 fold, H = 0.7166 ± 0.1734 fold; VEGF-C: L = 0.7266 ± 0.1574 fold, LE = 0.7757 ± 0.6490 fold, H = 0.8135 ± 0.1786 fold), the expression of VEGF-D was found to be significantly decreased across all three conditions (L = 0.4087 ± 0.2527 fold, LE = 0.2832 ± 0.1812 fold, H = 0.5376 ± 0.2769 fold).

As the local cytokine milieu may influence the integrity of the lymphatic and/or blood vessels, we next evaluated junctional permeability changes based on the gene expression of the Vascular Endothelial Growth Factors Receptor 2 and 3 (VEGF-R2 and VEGF-R3) as well as Connexin 43 (GJA1), Zonula occludens-1 (TJP1) and Claudin-5 (CLDN5) (Figure 3C,D). VEGF-R2 was significantly decreased across all conditions when compared to control tissue (VEGF-R2: L = 0.826 ± 0.247 fold, LE = 0.564 ± 0.192 fold, H = 0.407 ± 0.220 fold). Interestingly, the VEGF-R2 levels in lipohypertrophy were found significantly decreased, also in comparison to lipedema. VEGF-R3 expression was significantly increased only in secondary lymphedema when compared to all groups (VEGF-R3: L = 1.006 ± 0.299 fold, LE = 3.227 ± 1.067 fold, H = 1.406 ± 1.134 fold). GJA1 and CLDN5 were both significantly increased solely in secondary lymphedema, in comparison to both healthy control and lipedema tissue (GJA1: L = 0.999 ± 0.391 fold, LE = 3.132 ± 2.938 fold, H = 1.019 ± 0.596 fold) (CLDN5: L = 1.378 ±0.497 fold, LE = 4.576 ± 3.135 fold, H = 2.856 ± 2.883 fold) and in the case of GJA1, when compared to lipohypertrophy as well). No changes were detected in TJP1 expression levels (TJP1: L = 0.931 ± 0.254 fold, LE = 1.194 ± 0.526 fold, H = 1.061 ± 0.546 fold). 

### 2.4. Immune Cell Infiltrate Changes in Lipedema, Secondary Lymphedema and Lipohypertrophy Patients

**Figure 4:**
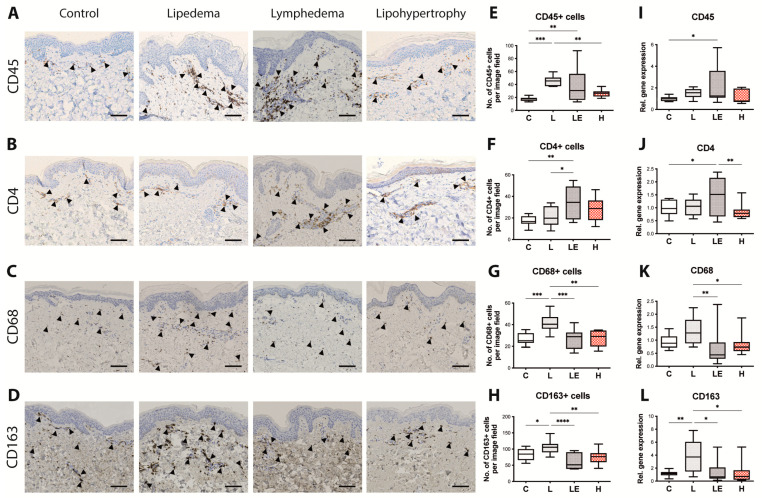
Increased but distinct immune cell presence in lipedema and secondary lymphedema, without signs of increased immune infiltration in lipohypertrophy. (**A**) CD45+ hematopoietic cell, (**B**) CD4+ T helper cell and (**C**) CD68+ macrophage (**D**) CD163+ macrophage staining of skin in control, lipedema, secondary lymphedema and lipohypertrophy paraffin sections (**E**–**H**) Quantification of histological analysis in tissue sections. An increased presence of CD45+ cells was detected in lipedema and secondary lymphedema but not in lipohypertrophy. Secondary lymphedema is characterized by an increased CD4+ infiltration, in comparison to both control and lipedema tissue. In lipedema an increased infiltration of both CD68+ and CD163+ cells in comparison to both the control and lipohypertrophy tissue is detected. (G-I) Relative gene expression (hereby control group = 1) of (**I**) CD45, (**J**) CD4, (**K**) CD68, (**L**) CD163. The findings here confirm most of the differences detected in the histological analysis. Black scale bar represents 100 μm. N = 10 in each group. Statistical significance is signified by asterisks (* *p* < 0.05, ** *p* < 0.01, *** *p* < 0.001, **** *p* < 0.0001).

As distinct immune components present a differentiating characteristic of both lipedema and lymphedema while the immune phenotype of lipohypertrophy is still vague, we next assessed the immune cell composition using tissue sections and gene expression analysis to quantify the presence of CD45+ cells (leukocytes), CD4+ cells (T helper cells), CD68+ cells (Macrophages) and CD163+ cells (M2-Macrophages) (Figure 4A–D). 

A significantly increased infiltration of CD45+ cells was detected in lipedema and secondary lymphedema tissue, while the presence of CD45+ cells in lipohyertrophy tissue was found comparable to healthy controls (C = 17.60 ± 3.148 cells/field, L = 45.68 ± 7.225 cells/field, LE = 39.49 ± 26.51 cells/field and H = 26.25 ± 5.003 cells/field). An increased infiltration of CD4+ cells was observed only in secondary lymphedema when compared to both the control and lipedema tissues, (C = 18.63 ± 3.405 cells/field, L = 21.48 ± 9.087 cells/field, LE = 34.86 ± 14.40 cells/field, H = 28.35 ± 11.05 cells/field). CD68+ and CD163+ macrophage cells were significantly increased only in lipedema (CD68: C = 27.09 ± 35.305 cells/field, L = 42.48 ± 8.662 cells/field, LE = 26.68 ± 9.210 cells/field and H = 27.29 ± 7.543 cells/field; CD163: C = 81.18 ± 19.04 cells/field, L = 104.5 ± 15.29 cells/field, LE = 64.03 ± 25.32 cells/field, H = 75.67 ± 22.28 cells/field) (Figure 4E–H).

Gene expression analysis conducted by qPCR confirmed most of these findings, indicating significantly increased CD45 expression (C = 1 ± 0.2219, L = 1.493 ± 0.4333, LE = 2.183 ± 1.814, H = 1.150 ± 0.6179) and CD4 expression (C = 1.006 ± 0.3018, L = 1.043 ± 0.3087, LE = 1.475 ± 0.7126, H = 0.8663 ± 0.2933) in secondary lymphedema compared to control tissue. The expression levels of CD4 in lipohypertrophy were found significantly decreased in comparison to secondary lymphedema as well. The expression of CD68 and CD163 was found significantly increased only in lipedema in comparison to secondary lymphedema and lipohypertrophy (CD68: L = 1.369 ± 0.4872 fold, LE = 0.6578 ± 0.6455 fold, H = 0.8341 ± 0.4209 fold; CD163: L = 3.808 ± 2.549 fold, LE = 1.399 ± 1.639 fold, H = 1.382 ± 1.183 fold) (Figure 4I–L), thus these findings are in line with our previous histological findings.

## 3. Discussion

Lipedema, secondary lymphedema and lipohypertrophy present three clinical entities characterized by the disproportional adipose tissue accumulation in one or more extremities and are commonly misdiagnosed or misinterpreted as obesity. While lipedema and secondary lymphedema have already been studied and compared to matching controls, so far they have not been assessed in comparison to each other and particularly versus lipohypertrophy, to accurately depict and classify their distinct histological and molecular characteristics. 

While several molecular and histological hallmarks in lipedema and secondary lymphedema have been established, thus far no data exists about defining features of lipohypertrophy. Therefore, for the purpose of this study we chose to analyze well-established defining features of lipedema and secondary lymphedema such as epidermal thickness, fibrosis, adipocyte size, changes in the lymphatic vasculature and immune cell infiltration in tissue samples from all four groups, namely lipedema, lipohypertrophy, secondary lymphedema and control tissue (Table 1).

Epidermal thickness is an important factor in secondary lymphedema, where the epidermis thickness increases with the severity of disease [7,23] and it is used as a primary outcome measure for the efficacy of lymphedema treatments, such as the anti-inflammatory drug ketoprofen [24] or anti-Th2 immunotherapy [25]. In recent studies, the increased epidermal thickness was also shown to be present in lipedema patients [4]. In both groups the results of the current study are aligning with already published data. Surprisingly, an increased epidermal thickness was not detectable in the lipohypertrophy group, separating it from lipedema and secondary lymphedema. 

As the increased adipose tissue deposition presents one of the most important clinical characteristics of all three pathologies, we next evaluated the adipocyte size and size distribution across the three conditions. Increased adipocyte size was detected in lipohypertrophy and lipedema in comparison to the control, but not in lymphedema. It is well-known that adipocyte hypertrophy is a hallmark of secondary lymphedema as well, but more pronounced in the later stages of disease. In the present study, secondary lymphedema patients from stages two and three were included, thus increasing the sample heterogeneity, which would explain why the increase in the adipocyte size was not significant compared to the control group. Furthermore, the vast majority of our samples (39 out of 40) were harvested from the leg, where the adipocyte enlargement might be less pronounced than in the arm [26]. The differences in the adipocyte size in comparison to the previous publication of our group [4] are attributed to the improvement of the quantification methodology using an improved automated algorithm (including more accurate exclusion of histology artifacts/doublets, etc.) and the adipocyte size of the control group remains in the normal range. Nevertheless, the well-established differences in the adipocyte size between the groups are present to the same extent [4].

In secondary lymphedema and lipedema the adipose expansion is accompanied by the remodeling of the extracellular matrix and fibrosis [27]. We evaluated this key histological characteristic by staining collagen in skin and fat sections using Sirius red and evaluating collagen content. Surprisingly, we detected significantly lower levels of fibrosis in the skin of lipohypertrophy patients in comparison to the controls, while the levels in the adipose tissue were found comparable to the controls. In secondary lymphedema the increased fibrosis was only detected in fat tissue, while in lipedema a strong trend towards increased adipose tissue fibrosis was noted, without reaching statistical significance. 

Due to the phenotypic similarities between secondary lymphedema and lipedema, a potential involvement of the lymphatic system in the onset or development of lipedema has been hypothesized. While in secondary lymphedema the modification of the lymphatic vascular architecture is an integral part of the disease`s pathomechanism, the impairment of the lymphatic system is discussed controversially in lipedema and has not been evaluated, to the best of our knowledge, in lipohypertrophy thus far. Interestingly, we identified significantly reduced cutaneous lymphatic vascular coverage in lipohypertrophy patients, when compared to all other groups. The absence of lymphatic vascular alterations in lipedema in regard to the number and size of the lymphatic vessels was further confirmed [5,6]. These findings also align with Al-Ghadban et al. [6] who was able to show that number and size of vessels do not significantly change in non-obese lipedema patients, although vessels are significantly larger in obese lipedema patients. Even though a change in lymphatic phenotype in the lymphedematous tissue would have been expected, this might have occurred due to the heterogeneity of our study cohort, whereby patients from different lymphedema stages were included. In the first stages of secondary lymphedema, a higher endolymphatic pressure leads to dilated lymphatic vessels. Hereby the constantly increased pressure leads to a compensatory increase in smooth muscle cells and fibrosis in later lymphedema stages, resulting in a functional decline and reduced lumen (and therefore size as well) of the lymphatic vessels. 

Genes associated with lymphangiogenesis and the junctional paracellular permeability of vessels such as GJA1 [28,29,30,31,32], CLDN5 [33,34] and VEGFR3 [35,36,37] were found upregulated only in secondary lymphedema, where true edema is present. In lipedema and lipohypertrophy the presence of edema has long been debated, with increasing evidence that the lymphatic system is not primarily defective. The significant decrease in VEGF-D expression observed in all diseased tissues aligns with the existing literature for lipedema [5] and secondary lymphedema [7] and presents a novel finding for lipohypertrophy. The decreased VEGF-D expression in secondary lymphedema might act as a compensatory mechanism for the increased vascular permeability and the increase VEGFR3 expression. Apart from the influence on the lymphatic vasculature, VEGF-D is known to promote a M1 macrophage polarization [38], whereas a decrease VEGF-D levels would be in favor of the well documented M2-macrophage infiltration in lipedema.

The distinct immune cell infiltration is a defining characteristic in both lipedema [4,5,6] and secondary lymphedema [7,8,9,10,11]. Recent studies have shown that an increased CD4+ T cell expression [8,11,39] is present both locally as well as systematically in secondary lymphedema patients [7]. Similarly, lipedematic tissue is characterized by the increased infiltration with CD68+ macrophages and predominantly M2 Macrophages (CD163+) [5,6]. Interestingly and in clear distinction to lipedema and secondary lymphedema, no increased immune cell infiltration was detected in lipohypertrophy, while the infiltration with CD4+, CD68+ or CD163+ cells was found comparable to the healthy controls.

The absence of an immune cell niche in lipohypertrophy, raises the question whether lipohypertrophy indeed presents a stage 0 lipedema or a distinct disease entity. If the hypothesis of lipedema developing from lipohypertrophy [1,2,12] is correct, the adipocyte hypertrophy increase would precede the infiltration of immune cells and particularly macrophages. This in turn would mean that possible stimuli for the attraction of cells could be coming from the hypertrophic adipocytes. On the other hand, macrophages are known to be pain mediators [40,41]. The main differentiating characteristic between lipohypertrophy and lipedema is the absence of pain of the affected extremities in lipohypertrophy and this characteristic seems aligned with the absence of a macrophage infiltration in lipohypertrophy. Further to that, the absence of the increased epidermal thickness along with the decreased levels of fibrosis and the reduced lymphatic vessel coverage in lipohypertrophy rather support the notion, that lipohypertophy presents a distinct entity. Further work is though necessary, focusing on the comparison of lipohypertrophy and stage one lipedema of larger populations, so that to evaluate the key molecular and histological determinants at the onset of lipedema versus lipohypertrophy. 

Based on the current data, it remains unclear whether lipohypertrophy could be considered as a normal variant of obesity. Our results indeed indicate that lipohypertrophy and obesity might share several characteristics, in particular regarding the adipose tissue expansion and architecture. In this study, all patient groups had a similar BMI in the range of overweight but not obese (defined as a BMI over or equal to 30 kg/m^2^); therefore, further studies are required to directly compare lipohypertrophy patients with obese patients.

A deeper understanding of the key determinants and mechanisms is important in all three pathologies, so that to accurately diagnose and monitor their development. Although a lot of progress has been conducted lately in better understanding the mechanisms underlying lipedema and secondary lymphedema, very little information is available for lipohypertrophy, that presents the most common differential diagnosis to lipedema. Although it is indeed briefly mentioned in some national lipedema guidelines as a differential diagnosis (German [13], UK [14], Dutch [15], USA [16]) it not widely considered a separate entity and little effort has been paid to define the histological and molecular landscape of the condition.

The study has been limited by its relatively small sample size attributed to the strict matching of tissues (gender, age, BMI, anatomical region), as well as the relative heterogeneity of the lipedema and secondary lymphedema populations, as patients from different stages have been included in the study. Despite that, the carefully selected patient cohorts with well-documented and validated diagnosis of the different disease entities as well as the consistent use of gender-, age-, BMI- and anatomically matched samples counterbalance the small group size and enabled the reproduction of various known hallmarks of the diseases studied. 

In conclusion, we were able to expand and establish key hallmarks of lipedema, lipohypertrophy and secondary lymphedema histologically and in gene expression. By comparing the histological and molecular determinants of all three diseases we were able to define characteristics distinguishing the conditions from one another. Further work should focus on the validation of our findings in a bigger study cohort and particularly on the comparison between lipohypertrophy and stage one lipedema. 

## 4. Materials and Methods

### 4.1. Study Population 

The studies involving human participants were reviewed and approved by Swiss ethics (BASEC-Nr.: 2019-00389) and Ethical Committee of the University Hospital Goettingen, State of Lower Saxony, Germany (Nr. 23-11-17). All study participation was conducted under the principles of the Declaration of Helsinki and informed consent was given by all patients preceding the beginning of the study. Gender-, age-, BMI and anatomical location-matched probes were collected from patients with secondary lymphedema, lipedema, lipohypertrophy as well as healthy control patients (Table 2).

#### 4.1.1. Diagnostic Criteria

##### Lymphedema

The diagnosis of lymphedema was placed by two independent consultant physicians, a plastic surgeon and an angiologist. All secondary lymphedema patients developed lymphedema upon lymphadenectomy and only female patients were included in this study. The diagnostic criteria included: pathologic micro-lymphangiography (conducted by the angiologist), localized pitting edema, positive Stemmer sign, increased circumferences of the affected extremity in comparison to the contralateral side, no response or continuing progression of the disease despite conservative treatment.

##### Lipedema

Lipedema was diagnosed by two independent consultant physicians, a plastic surgeon and an angiologist. The diagnosis was based on the criteria of Wold et al. [42], whereby all included patients fulfilled all criteria. They were all women with a bilateral adipose tissue increase of the legs that persisted weight loss or elevation of limbs whereby feet were excluded and the Stemmer sign was negative. Distinctive lipedema features were present, namely pain, tenderness and a tendency to bruise. 

##### Lipohypertrophy

For the diagnosis of lipohypertrophy we chose the same criteria as in lipedema, excluding pain or discomfort in the affected extremities.

### 4.2. Tissue Collection

The tissue was obtained during surgical procedures using sharp surgical dissection (scalpel). The samples were deriving from the ventral aspect of the lower extremities at the level superficial to the Scarpa fascia (39/40) or the dorsolateral aspect of the arm (1/40) at the level of the superficial adipose tissue. The tissue collection method was identical in both study centers. Samples for histology and immunohistochemistry analysis were fixed in 4% paraformaldehyde in phosphate-buffered saline, dehydrated and subsequently paraffin embedded. Fat tissue for RNA extraction was frozen in liquid nitrogen.

### 4.3. Histology and Immunohistochemistry Staining

Paraffin embedded tissues were cut into 5 μm sections and stained at the Center for Surgical Research at the University Hospital of Zurich according to previously published protocols [4]. Therefor sections were deparaffinized and rehydrated. For the histology analysis Hematoxylin/Eosin and Sirius red stains was performed. 

For immunohistochemical stains of CD45 (monoclonal mouse anti human, IR751, Dako, Agilent, Santa Clara, CA, USA), CD68 (monoclonal mouse anti human, IR613, Dako, Agilent), CD4 (monoclonal mouse anti human, IR649, Dako, Agilent) and CD163 (monoclonal rabbit antihuman, abcam ab 182422; 1:300), Target Retrieval Solution—high pH9.0—and for stainings with Podoplanin (monoclonal mouse anti human, IR072, Dako, Agilent), Target Retrieval Solution—high pH6.0—were used for antigen retrieval, subsequently endogenous peroxidase activity was blocked using 3% hydrogen peroxide (Merck). Following blocking with goat serum, the sections were incubated with the primary antibodies in RTU dilution or otherwise mentioned dilution. Bound antibodies were visualized using HRP coupled secondary antibodies and DAB substrate (Dako K3468) following the according guidelines. 

### 4.4. Immunohistochemistry Analysis

Histology images were acquired using the Zeiss Axio Scan Z1, equipped with a Hitachi HV-F202FCL camera and thereafter scanned using the Plan Apochromat 20×/0.8 numerical aperture objective. 

The Analysis was conducted using five randomly placed Regions of Interest (ROIs) and averaging out the outcomes to obtain conclusions. For the SR evaluation, ImageJ Software was used by performing an analysis with fixed color thresholds and assessing the stained percentage of the ROI. 

### 4.5. RNA Extraction and Quantitative Polymerase Chain Reaction (qPCR)

RNA from adipose samples from secondary lymphedema, lipedema, lipohypertrophy and control patients was isolated and purified using RNeasy Lipid Tissue Mini Kit (Qiagen, Hilden, Germany) following the manufacturer’s instructions. Complementary DNA was transcribed from a 500 ng RNA template, using the high-capacity cDNA Reverse Transcription Kit (ThermoFisher Scientific, Waltham, MA, USA). Quantitative PCRs were then performed using SYBR Green Master Mix (appliedbiosystems, Waltham, MA, USA) and B2M as the Housekeeping Gene. Primers (Microsynth, Balgach, Switzerland) are provided separately (see Table S1). qPCRs were carried out using QuantStudio (v1.4.2) Software in which ∆∆CT method was used to calculate the fold changes of gene expression.

### 4.6. Statistics 

The statistical analysis was performed using GraphPad Prism V 8.0 (GraphPad Software, San Diego, CA, USA). All data represent the mean ± SD, as depicted in whisker plots exhibiting the 5–95 percentiles. For the comparison of all four samples, a one-way ANOVA followed by a Tukey test was performed. *p* < 0.05 was accepted as statistically significant.

## Figures and Tables

**Table 1:** Disease Characteristics adapted from German [13], UK [14], Dutch [15], USA [16] guidelines and completed with findings from the present study (with grey background).

	Lipedema	Secondary Lymphedema	Lipohypertrophy
Sex	Women	Women and Men	Women
Family History	+	-	+
Bilateral Swelling	+	-	+
Symmetric Swelling	+	-	+
Disproportion	+	+	+
Edema	-/(+) *	+++	-
Inclusion of Feet	-	+	-
Pain	+	+	-
Bruising Tendency	+	-	-
Tissue Turgor	soft	firm	soft
Affinity to Infection	-	+	-
Epidermal Thickness	++	++	(+)
Adipocyte Size	+++	+	+++
Lymphatic system	unchanged phenotype	lymphatic impairment	decreased lymphatic coverage
Fibrosis	+	+++	-
CD45+ cells	+++	++	-
CD4+ T helper cells	-	+++	-
CD68+ Macrophages	+++	-	-
CD163+ Macrophages	+++	-	-

Explanation of symbols: + to +++ present; (+) possible; - not present. * Edema is only present in stage 4 (lipo-lymphedema).

**Table 2:** Patient Characteristics.

	Control	Lipedema	Lipohypertrophy	Lymphedema
Number of cases	10	10	10	10
Gender				
Female	10	10	10	10
Male	0	0	0	0
Average BMI	27.86 ± 4.478	29.10 ± 3.816	27.62 ± 4.943	26.55 ± 4.815
Average Age	48.45 ± 9.501	50.56 ± 11.41	46.20 ± 16.36	60.70 ± 11.15
Lymphedema Stage				
Stage 1				
Stage 2				7
Stage 3				3
Lipedema Stage				
Stage 1				
Stage 2		6		
Stage 3		4		
Harvesting location Lower extremity	10	10	10	9
Upper extremity				1

## Supplementary Materials

The following supporting information can be downloaded at: https://www.mdpi.com/article/10.3390/ijms24087591/s1.
