# Supplementary material for: A Comparative Analysis to Dissect the Histological and Molecular Differences among Lipedema, Lipohypertrophy and Secondary Lymphedema
Source: Int J Mol Sci. 2023 Apr 20;24(8):7591. doi: 10.3390/ijms24087591 (PMC10144050; doi:10.3390/ijms24087591)
Supplement: Supplementary file 1 [file ijms-24-07591-s001.zip › ijms-2328446-supplementary.pdf]

Suppl. Table S1. Primer Sequences

Primer	Sequence (5′ to 3′)
B2M forward	TGTGCTCGCGCTACTCTCTCT
B2M reverse	CGGATGGATGAAACCCAGACA
VEGF A forward	CTACCTCCACCATGCCAAGT
VEGF A reverse	GCAGTAGCTGCGCTGATAGA
VEGF C forward	CACCACCAAACATGCAGCTG
VEGF C reverse	TGAAAATCCTGGCTCACAAGC
VEGF D forward	ATGGACCAGTGAAGCGATCAT
VEGF D reverse	GTTCCTCCAAACTAGAAGCAGC
VEGF-R2 forward	GGCCCAATAATCAGAGTGGCA
VEGF-R2 reverse	TGTCATTTCCGATCACTTTTGGA
VEGF-R3 forward	TCTGCTACAGCTTCCAGGTGG
VEGF-R3 reverse	GCAGCCAGGTCTCTGTGGAT
CLDN5 forward	GGGAACTTCCTGAAGTGG TGT
CLDN5 reverse	TCCCATGGCAAACAGAGAGG
TJP1 forward	GGAGAGGTGTTCCGTGTTGT
TJP1 reverse	GAGCGGACAAATCCTCTCTG
GJA1 forward	GGAGATGAGCAGTCTGCCTTTC
GJA1 reverse	TGAGCCAGGTACAAGAGTGTGG
CD4 forward	CCTCCTGCTTTTCATTGGGCTAGG
CD4 reverse	TGAGGACACTGGCAGGTCTTCT
CD45 forward	CTTCAGTGGTCCCATTGTGGTG
CD45 reverse	CCACTTTGTTCTCGGCTTCCAG
CD68 forward	CGAGCATCATTCTTTCACCAGCT
CD68 reverse	ATGAGAGGCAGCAAGATGGACC
CD163 forward	ACATAGATCATGCATCTGTCATTTG
CD163 reverse	ATTCTCCTTGGAATCTCACTTCTA
